# Inhibitory effects of evodiamine on zymosan-induced inflammation: inactivation of NF-κB by inhibiting IκBα phosphorylation

**DOI:** 10.1186/cc14014

**Published:** 2014-12-03

**Authors:** X Fan, J-y Zhu, Z Liu, J Yan, Q Ma, H-p Liang

**Affiliations:** 1 State Key Laboratory of Trauma, Burns and Combined Injury, Research Institute of Surgery, The Third Military Medical University, Chongqing, People's Republic of China

## Introduction

Inflammation is a host defense reaction against pathogenic infection. In this process, inflammatory cytokines contribute to combat against infection, but excess cytokines will lead to tissue damage. Nonsteroidal anti-inflammatory drugs and corticosteroids are commonly used for regulating these inflammatory mediators and treatment of inflammatory disorders. But these drugs are not sufficient for clinical practice due to their adverse effects, so new anti-inflammatory drugs are still needed. Evodiamine (EVO), an important alkaloidal component extracted from the fruit of Evodiae fructus, possesses the property of analgesia, antiemesis, and vascular dilatation. Its anti-inflammatory effect and underlying mechanism were investigated using a zymosan-induced inflammatory model.

## Methods

*In vitro*, RAW264.7 cells and primary peritoneal macrophages were treated with different doses of EVO (25, 50 or 100 μM) for 1 hour prior to incubation with zymosan (100 μg/ml), and the effects of EVO on protein and mRNA levels of proinflammatory cytokines were determined by ELISA and qRT-PCR, respectively. *In vivo*, peritonitis was induced in C57BL/6 mice by zymosan (500 mg/kg) injection, and the effects of EVO (10 mg/kg) on plasma cytokine levels and organ injury were evaluated. Activation of the NF-κB signal pathway was investigated by ELISA-based Trans-AM transcription factor NF-κB p65 kit, immunocytochemistry and western blotting.

## Results

EVO effectively suppressed the expression of IL-1β, IL-6 as well as TNFα in both protein and mRNA level *in vitro*. It can also attenuate zymosan-induced DNA-binding activity of NF-κB, which was achieved through the inhibitory effects on the phosphorylation of inhibitory kappa B α (IκBα) and p65 nuclear translocation, but there was little association with mitogen-activated protein kinase activation. *In vivo*, treatment with EVO could ameliorate inflammatory cell infiltration and vascular ectasia induced by zymosan in both lung and intestine tissues. EVO can markedly decrease the level of TNFα and IL-6 in plasma, and effectively downregulate expression of IL-6, TNFα and myeloperoxidase in both lung and intestine. Moreover, cell apoptosis in organs was also attenuated by treatment of EVO. The underlying mechanism that a decrease in the phosphorylation of IκBα and the subsequent transcription activity of NF-κB was also confirmed.

## Conclusion

Taken together, our data demonstrate that EVO displays anti-inflammatory actions *in vitro *and *in vivo *by suppressing the phosphorylation of IκBα and inactivating NF-κB, which suggests that EVO is a potential therapeutic agent against inflammatory disorder.

